# Traffic flow prediction via dynamic hypergraph learning

**DOI:** 10.1371/journal.pone.0347846

**Published:** 2026-04-28

**Authors:** SiWei Wei, Yang Yang, ChunZhi Wang

**Affiliations:** 1 School of Computer Science and Artificial Intelligence, Wuhan University of Technology, Wuhan, China; 2 CCCC Second Highway Consultants Co.Ltd., Wuhan, China; 3 Hubei Key Laboratory of Transportation Internet of Things(Wuhan University of Technology), Wuhan, China; 4 School of Computer Science, Hubei University of Technology, Wuhan, China; National University of Defense Technology, CHINA

## Abstract

In the field of intelligent transportation systems, efficiently and accurately predicting traffic flow and its evolution trends has become an important and urgent research task. Graph neural networks have been widely used in traffic flow prediction problems, but many methods ignore the high-order relationship of patterns between traffic flow and traffic nodes. To address the above issues, we propose a Transformer-based Hypergraph Convolutional Network (TSHGCN) for traffic flow prediction. Firstly, we adopt a hypergraph structure to more effectively capture the high-order nonlinear spatial correlations between traffic nodes. Then, an improved Transformer network is proposed, which accurately captured the global temporal features among various traffic nodes by combining the time distillation mechanism and the self-attention network. In addition, we integrate the above spatiotemporal modeling through efficient channel attention mechanism and multi-scale temporal information fusion mechanism, accurately extracting spatiotemporal features and achieving the final refined representation of traffic flow. Experiments on the California datasets (PeMSD4 and PeMSD8) with 5 independent random seed runs and strict statistical tests show that the TSHGCN model achieves the best performance on core metrics (MAE, RMSE, MAPE) under a unified experimental setting, and the performance improvement over state-of-the-art baselines is statistically significant.

## Introduction

The rapid development of the Internet of Things (IoT), big data, and artificial intelligence (AI) has not only significantly enhanced the capability of collecting and processing traffic data but also accelerated the intelligent development of public transportation. As a core technology in intelligent transportation systems, traffic flow prediction has been extensively researched and applied both domestically and internationally in recent years. With the rapid advancement of AI and big data technologies, traffic flow prediction methods have continuously evolved and progressed, transitioning from traditional statistical models to sophisticated machine learning and deep learning techniques. Researchers both domestically and internationally have made remarkable progress in this field.

Early scholars widely applied traditional statistical methods such as the Autoregressive Integrated Moving Average (ARIMA) model and exponential smoothing, which are particularly suitable for short-term traffic flow prediction due to their simplicity of implementation and high computational efficiency. With the development of machine learning technology, methods such as Support Vector Machines (SVM) and Artificial Neural Networks (ANN) have gradually been introduced, especially deep learning models such as Recurrent Neural Networks (RNN) and Long Short-Term Memory Networks (LSTM), which excel in processing time series data and can capture nonlinear and long-term dependencies in data, gradually becoming a popular choice for traffic flow prediction. In addition, domestic scholars have also attempted to integrate multiple methods, such as combining statistical methods with machine learning methods or integrating the prediction results of multiple models, to improve prediction accuracy and robustness. Subsequently, scholars have developed more complex models based on traditional statistical models, such as Dynamic Linear Models (DLM) [1] and Generalized Additive Models (GAM) [2], which provide more flexible modeling capabilities in dealing with nonlinear relationships and temporal variations. With the rapid development of new-generation information technology, machine learning and deep learning technologies have been widely applied in fields such as computer vision and data prediction [3–8]. For example, foreign scholars have attempted to use deep learning architectures such as Deep Belief Networks (DBN) [9] and Stacked AutoEncoders (SAE) [10], which excel in processing large-scale data and complex patterns. Beyond architectural advancements, hybrid approaches that combine optimization algorithms with deep learning models have also shown significant promise in enhancing forecasting performance. For instance, Sajja et al. successfully integrated the Mayfly Algorithm with a Bidirectional Gated Recurrent Unit (BiGRU) [11] to optimize model parameters for retail supply chain forecasting, demonstrating that such synergistic methods can lead to more accurate and robust predictions. This aligns with the overarching goal of our work—to improve predictive accuracy through innovative model design, albeit from an architectural integration perspective rather than a parameter optimization one.

Graph Neural Networks (GNNs) have also received extensive attention in research. In traffic flow prediction tasks, Recurrent Neural Networks (RNNs) [12] and their variant Long Short-Term Memory Networks (LSTMs) are widely used to effectively process time series data and capture long-term dependencies. Meanwhile, Convolutional Neural Networks (CNNs) are also employed to process spatial data, effectively extracting spatial features from graph data and predicting the spatial distribution of traffic flow more accurately. For example, the CNN-GRU [13] model uses volume feature sequences as input to the GRU, which effectively addresses the vanishing and exploding gradient problems that may occur during long sequence training through a variation of the Long Short-Term Memory network. Furthermore, STATF [14] combines an LSTM encoding layer and an LSTM decoding layer, introducing an attention mechanism that adaptively learns the spatiotemporal dependencies and nonlinear correlation features of urban traffic flow data, achieving more accurate urban traffic flow prediction. However, these models may have some flexibility and timeliness issues in modeling graph data and adapting to long-term trend changes.

In recent years, spatiotemporal graph neural networks have become a research hotspot in the field of traffic flow prediction. These deep learning networks treat traffic data as a dynamic spatiotemporal network and model it using graph neural network techniques, including spatiotemporal modeling, spatiotemporal sequence modeling, and spatiotemporal attention mechanism modeling. For example, the T-GCN model combines Graph Convolutional Networks (GCN) and Gated Recurrent Units (GRU) to capture spatial and temporal dependencies and solve spatiotemporal traffic prediction problems. DCRNN [15] introduces graph convolutional networks to predict spatiotemporal data and uses Diffusion Graph Convolutional Networks to describe the diffusion process of information in spatial networks, thereby performing traffic flow prediction tasks based on graph structure and time series data. STGCN [16] utilizes CNN to model temporal correlation and GCN to model spatial correlation. Graph Wave Net [17] proposes an adaptive adjacency matrix to capture hidden spatial dependencies accordingly. This matrix can automatically discover invisible graph structures from data without any prior knowledge guidance. It also proposes an effective framework to simultaneously capture spatiotemporal correlation, specifically by combining graph convolution and dilated causal convolution. STSGCN [18] constructs a local spatiotemporal graph and utilizes a spatiotemporal synchronous graph convolution module to capture local spatiotemporal correlation. ASTGCN [19] utilizes a spatiotemporal attention mechanism to capture the dynamic spatiotemporal correlation of traffic data, combines a simple traffic network structure, extracts spatial features using graph convolution, and describes dependency relationships using convolution in the time dimension. GMAN [20] adopts an encoder-decoder architecture composed of stacked spatiotemporal attention blocks, each of which includes a spatial attention mechanism for modeling dynamic spatial correlation, a temporal attention mechanism for modeling nonlinear temporal correlation, and a gated fusion mechanism for adaptive fusion of spatial and temporal representations. The attention mechanism between the encoder and decoder models the direct relationship between historical time steps and future time steps, which helps mitigate the impact of error propagation. Transformer has demonstrated excellent effectiveness in natural language processing tasks and has been successfully applied to computer vision tasks. Due to its superior performance, it is widely used in various fields. MTGNN [21] automatically extracts unidirectional relationships between variables through a graph neural network learning module, and further proposes novel hybrid skip-propagation layers and dilated initial layers to capture spatial and temporal dependencies in time series.

Despite significant advancements in technology in the field of traffic flow prediction, there are still a series of key challenges and inherent limitations. Existing road-based network graphs typically only represent pairwise connections between nodes, making it difficult to effectively model the high-order dependencies between sensor nodes in the traffic network. This apparent limitation directly results in the model’s inability to fully capture the complexity and dynamically changing spatial characteristics of traffic data.

Not only that, although existing models have remarkable performance in time series modeling, they often struggle to accurately capture the complex long-term time dependencies and multi-scale temporal characteristics in traffic flow. Due to the lack of efficient decomposition and modeling capabilities for time series, existing methods perform poorly in dealing with complex temporal patterns. Some existing models attempt to capture long-term time dependencies and spatial dependencies through complex structures, but their model designs are overly complicated, resulting in limited performance improvements in practical applications. This, in turn, makes it difficult to achieve an ideal balance between computational cost and prediction accuracy.

To effectively address these issues, we innovatively proposes a Transformer-based hypergraph convolutional neural model (TSHGCN) for traffic flow prediction. The contributions of this model are as follows:

Firstly, an efficiently designed channel attention mechanism (ECA) is adopted in this model. This mechanism can flexibly adjust the weights of different information channels based on actual spatiotemporal data, greatly enhancing the model’s ability to predict traffic flow data without significantly increasing computational costs.

Secondly, in terms of spatial modeling, this model designs a spatial feature learning method based on hypergraph convolution, which takes into account the multi-node interaction characteristics of the transportation network and enhances the modeling capability for non-Euclidean spatial structures. This method constructs a general graph into a hypergraph structure, calculates the node degree matrix and hyperedge degree matrix based on this, constructs the Laplacian matrix of the hypergraph, and finally performs hypergraph convolution to achieve effective diffusion and aggregation of features in transportation nodes. Compared with the traditional Graph Convolutional Network (GCN), the hypergraph convolution method can more comprehensively and accurately model the spatial topological structure of the transportation network and improve the ability to capture complex spatial dependencies.

Thirdly, in terms of temporal learning, to effectively capture the long-term temporal dependencies in traffic flow, this model draws inspiration from the Informer self-attention mechanism and distillation operation, and simultaneously introduces modeling methods for recent, daily, and weekly cycles. An improved Transformer has been carefully designed. This improvement enables the model to efficiently process long-sequence temporal data and accurately capture multi-scale temporal features, such as short-term fluctuations and long-term trends.

In addition, the model proposed in this paper adopts a modular design concept, efficiently integrating ECA, hypergraph convolution, and Transformer. By integrating these modules into a spatial and temporal learning module, this design significantly reduces computational complexity while ensuring high performance of the model.

Finally, the experimental results clearly demonstrate that the model proposed outperforms most existing methods across the board on real-world datasets, providing an efficient and powerful solution for traffic flow prediction tasks.

## Related works

**Fig 1:**
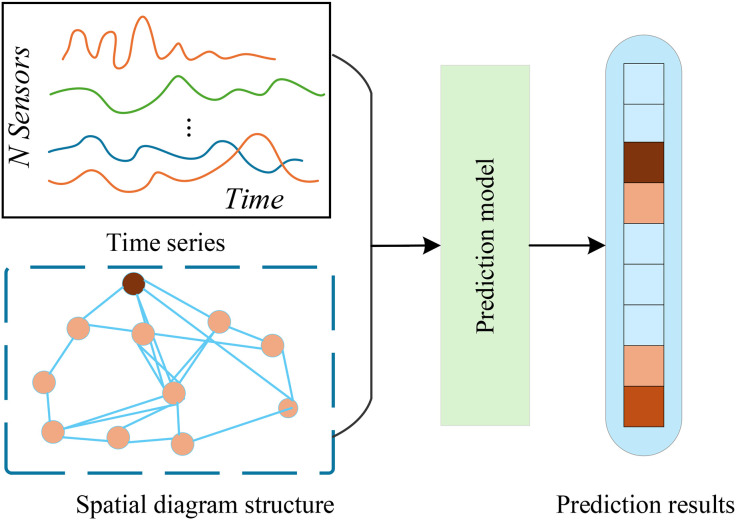
Spatio-temporal network prediction task.

The core task of traffic flow prediction is to accurately capture the complex spatiotemporal dependencies in traffic data, in order to precisely prediction key indicators such as traffic flow, operating speed, and congestion status for a specific future time period. Research in this field integrates cutting-edge achievements from multiple disciplines, including traffic flow theory, statistics, machine learning, and deep learning. Its technical approaches have gradually evolved from traditional time series analysis methods to deep spatiotemporal modeling methods based on graph neural networks, Transformer architectures, and multimodal fusion. The difficulties of traffic flow prediction mainly stem from the complexity of its spatiotemporal coupling. As shown in Fig 1, traffic data not only exhibits significant temporal dynamic characteristics such as periodic peaks and valleys, long-term trend changes, and sudden fluctuations, but is also constrained by the spatial topological structure of the road network. For example, the congestion propagation effect between adjacent road segments may lead to rapid changes in local traffic conditions, affecting surrounding areas. Traditional prediction methods, such as autoregressive integrated moving average models and Kalman filtering algorithms, although having certain advantages in capturing temporal characteristics, often fall short in dealing with complex nonlinear spatiotemporal relationships.

In recent years, deep learning methods have gradually become a research hotspot due to their powerful spatiotemporal modeling capabilities. Taking spatiotemporal graph neural networks as an example, this method greatly improves the performance of traffic flow prediction by encoding and modeling road network topology and time series dependencies. However, when faced with data scarcity, noise interference, and anomalies caused by unexpected events, this method still needs to further enhance its generalization ability. Although current research has made significant progress in prediction accuracy, computational efficiency, and application fields, there are still many challenges that provide ample room for future research

### Efficient channel attention

The development of channel attention mechanisms can be traced back to the initial explorations in the field of computer vision. In 2018, researchers first proposed the complete channel attention mechanism, Squeeze-and-Excitation Networks (SENet) [22]. SENet pioneered the design of a two-stage Squeeze-Excitation processing flow: in the Squeeze stage, the spatial features of each channel are compressed into a scalar through global average pooling; in the Excitation stage, a bottleneck structure consisting of two fully connected layers is employed to learn the nonlinear relationships between channels, and the Sigmoid function is used to generate the weight coefficients for each channel. This mechanism enables the network to adaptively recalibrate channel feature responses, achieving significant performance improvements on ImageNet classification tasks. However, SENet has two notable limitations: firstly, the number of parameters in its fully connected layers is squared with the number of channels, leading to high computational costs when dealing with high-dimensional features; secondly, this method completely ignores the possible local correlations between adjacent channels when modeling channel relationships. These shortcomings are particularly evident when dealing with high-resolution images or video data. To address the deficiencies of SENet, subsequent research has proposed various improvement schemes. For example, the Convolutional Block Attention Module (CBAM) [23] combines channel attention and spatial attention in series, supplementing the deficiencies of channel attention by introducing spatial attention. Although CBAM performs well on multiple visual tasks, it essentially continues the channel attention design idea of SENet and fails to fundamentally solve the computational efficiency problem. Furthermore, CBAM requires additional computation for spatial attention, further increasing model complexity. Another important development direction of attention mechanisms is lightweight design. The Efficient Channel Attention mechanism ECA-Net [24] (Efficient Channel Attention) uses one-dimensional convolution to replace fully connected layers to establish local cross-channel interactions, significantly reducing computational complexity while maintaining excellent performance. This improvement makes it particularly suitable for processing sequential data with spatiotemporal characteristics. For example, efficient channel attention mechanisms are actively being utilized in the field of traffic flow prediction, becoming a core technology for improving the accuracy and efficiency of spatiotemporal network traffic flow prediction tasks. This mechanism can adaptively adjust the importance of different data channels, providing a new solution direction for modeling complex spatiotemporal features. Traffic flow data has particularly high spatiotemporal complexity. Spatially, there are complex spatial correlations between special traffic nodes such as intersections and busy road segments at different geographical locations. In terms of time, traffic flow exhibits dynamic changes such as periodicity, trend, and suddenness, exhibiting a rapidly changing characteristic. Traditional spatiotemporal network prediction models struggle to effectively focus on key information channels, limiting the predictive power of the model.

The core advantage of the efficient channel attention mechanism lies in its ability to dynamically adjust the weights of channel features. At the spatial level, it identifies key channels that describe the spatial correlation of traffic nodes, such as those reflecting the interaction of major road traffic flows, enhances their features, marks and suppresses unimportant channels, and helps the model accurately understand the spatial structure of the traffic network. At the temporal level, this mechanism performs exceptionally well. The characteristics of traffic volume vary greatly across different time periods. For example, there is commuting traffic from residential areas to commercial areas during the morning peak. The channel attention mechanism can focus on channel features that affect traffic flow changes during specific time periods, assign higher weights, and improve the prediction accuracy for the corresponding time periods.

**Fig 2:**
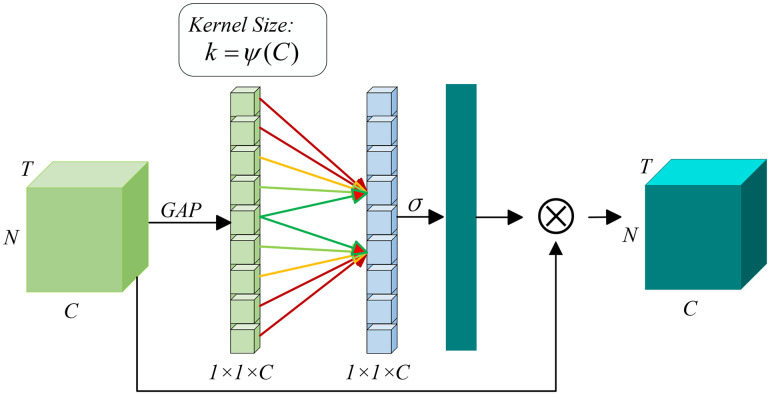
Efficient Channel Attention Mechanism.

In terms of technical implementation, as shown in Fig 2, the efficient channel attention mechanism generally performs global average pooling on the input spatiotemporal features first, compressing the channel feature information into scalars to obtain global information. Then, it applies a fully connected network to perform nonlinear transformation on these scalars to generate attention weights. Finally, the weights are multiplied by the original channel features to achieve adaptive adjustment. Experiments in this paper show that the model incorporating this mechanism has higher accuracy and stability in traffic flow prediction, with significant advantages over traditional methods.

### Graph Neural Networks

In traffic flow prediction tasks, Graph Neural Networks (GNNs) have become a research hotspot due to their excellent performance in spatiotemporal networks. As the core component of GNNs, graph convolution can effectively capture the spatial features of traffic networks and combine them with temporal information to achieve accurate predictions. A traffic network is a complex graph structure with intersections as nodes and roads as edges. When dealing with simple graph structures, graph convolution learns spatial dependencies by collecting information from nodes and their first-order neighbors. This local information aggregation method can effectively depict the direct correlation between nodes. The traffic volume at a certain intersection in a city is influenced by its own historical data and the traffic conditions of surrounding intersections. The Generalized Convolutional Network (GCN) uses a symmetrically normalized Laplacian matrix to perform weighted summation on the features of nodes and their adjacent nodes, with weights reflecting the degree of correlation between nodes. Through this simple graph-based modeling approach, the aggregation of information from adjacent nodes can better simulate traffic flow states, providing a basic spatial dependency modeling for traffic prediction.

Traditional graph structures are primarily modeled based on binary relations, representing transportation networks through simple graphs composed of nodes and edges. In this representation method, edges can only connect two nodes, describing pairwise relationships between nodes. Although this simple graph structure has high computational efficiency, it has obvious limitations in depicting real-world traffic scenarios: firstly, it cannot effectively model high-order interaction relationships between multiple intersections; secondly, edge weights are usually preset statically, making it difficult to reflect dynamically changing traffic conditions; finally, traditional graph structures often ignore the spatiotemporal heterogeneity characteristics in the process of traffic flow propagation. These limitations have prompted researchers to explore more advanced graph representation methods.

The innovative design of graph structures provides new ideas for improving the performance of graph convolution. In real-world road networks, traffic flow propagation resembles a diffusion phenomenon, where vehicle movements affect the traffic conditions at adjacent intersections. The graph diffusion method [25] establishes a node diffusion relationship graph through random movement or diffusion of the graph. By using this graph, graph convolution can more accurately simulate the flow propagation process, providing rich spatial topological information for the model and improving prediction accuracy. Hypergraph is an extended format that can connect multiple nodes to one edge, thus representing multiple relationships more accurately. Multiple intersections interacting near large commercial areas can be represented by hyperedges in the hypergraph concept. These innovative graph structure modeling methods have brought new breakthroughs to traffic flow prediction.

### Transformer

The Transformer architecture [26] was initially proposed by scholars in the field of machine translation in 2017. Its core innovation lies in constructing a deep neural network entirely based on a self-attention mechanism, abandoning traditional recurrent neural network and convolutional neural network structures. The main advantages of Transformer are reflected in three aspects: firstly, its parallel computing characteristics significantly enhance model training efficiency; secondly, the multi-head attention mechanism enables simultaneous attention to information at different positions in the sequence; finally, the introduction of positional encoding effectively preserves the sequential information of the input sequence. These characteristics make Transformer particularly suitable for processing sequence data with long-range dependencies.

In the task of spatiotemporal network traffic prediction, time series prediction is a crucial step. The introduction of the Transformer mechanism has brought about a significant breakthrough in this field, as traffic flow data is essentially a complex time series. Its values at different time points are not isolated but contain rich temporal dependencies. These relationships include short-term fluctuations, such as changes in traffic flow at different times within an hour, as well as long-term trend characteristics, such as differences in traffic patterns between weekdays and weekends. Traditional time series prediction methods often struggle to fully capture these complex multi-level dependencies.

The Transformer mechanism, with its powerful self-attention mechanism, has become an effective tool for solving this problem. The self-attention mechanism enables the model to dynamically calculate the correlation between each time step in the sequence and all other time steps when processing time series, thereby assigning corresponding attention weights to each time step. This feature plays an important role in predicting traffic flow time series. For example, when predicting traffic flow at a certain moment, the model can use the self-attention mechanism to identify which past time points of traffic data are most critical for predicting the current moment. During the morning rush hour, changes in traffic flow from the previous hour or even earlier may have a significant impact on the current traffic flow. The self-attention mechanism can assign higher weights to these relevant time steps, allowing the model to pay more attention to these key information and improve the accuracy of prediction. With the above functions, the Transformer mechanism provides more effective assistance for spatiotemporal network traffic prediction.

## Methods

This model abstractly describes the traffic network as an undirected graph with a topological structure *G* = < *V*, *E*, *A* > , where *V* is the set of nodes in the graph *G*, and each traffic graph is equipped with *n* sensors to collect traffic information; *E* is the set of edges connecting the various nodes *G*, and the adjacency matrix of the graph is denoted by $A\in R^{N\times N}$, This undirected graph is composed of both the node set *V* and the edge set *E*. On this basis, in order to more accurately depict the complex relationships of the traffic network, this model performs a structural-level hypergraph construction operation on the undirected graph *G*, ultimately obtaining a hypergraph $\tilde{G}$.

Historical traffic signals can be represented as $X=R^{T\times N\times C}$, which stands for the observed values of all sensors in the traffic network over the past *T* time steps. To conduct traffic flow prediction more effectively, this paper proposes a Transformer-based Traffic Flow Prediction Model with Hypergraph Convolution (TSHGCN). This network draws on the modeling ideas of the MCSTGCN network, focusing on the recent, daily, and weekly periodic information of the prediction target. Assuming the current time is *t*_0_ and the length of the *t*ime window to be predicted is *T*_*p*_, three time series segments with lengths of *T*_*h*_, *T*_*d*_, and *T*_*w*_ are extracted along the time axis, serving as the input components for the recent, daily, and weekly periodic inputs in the model, respectively. These three time series segments are as follows:

The recent segment $X_{h}\in R^{T_{h}\times N\times C}$ selection involves selecting historical time series segments that are closely adjacent to the prediction period, aiming to depict the direct impact of the traffic state at the previous moment on the traffic at the next moment at a single node, providing a basis for the model to capture the short-term dynamic changes in traffic flow.

The daily cycle segment $X_{d}\in R^{T_{d}\times N\times C}$ is composed of sequence segments that fully correspond to the prediction target period within a certain number of days prior to the prediction period. Its function lies in mining periodic patterns that occur cyclically in traffic data with days as the basic unit, enabling the model to learn similar patterns of daily traffic flow changes within the same time period.

The weekly period segment $X_{w}\in R^{T_{w}\times N\times C}$ involves selecting sequence segments from several weeks prior to the prediction period that not only share the same time period as the prediction target but also have consistent week attributes. This aids the model in identifying systematic patterns of change in traffic data on a weekly basis, fully considering the impact of different weeks on traffic flow.

After extracting the aforementioned three types of periodic information, they undergo weighted input processing. Simultaneously, by learning parameters and dynamically adjusting the relative importance of each component in the prediction task, the required input traffic signals for the entire model are ultimately generated $X_{wdh}=(w⊙X_{w}+d⊙X_{d}+h⊙X_{h})\in R^{(wT_{w}+dT_{d}+hT_{h})\times N\times C}$, enhancing the model’s accuracy and adaptability in predicting traffic flow.

The TSHGCN model proposed in this paper utilizes *n* traffic flow data from historical time periods *T* for each node in the transportation network structure $X_{n}^{T}=(x_{1},x_{2},\cdots ,x_{T})\in R^{(wT_{w}+dT_{d}+hT_{h})\times N\times C}$ to achieve accurate prediction of traffic flow data for future time periods *P*. If we represent this model as a prediction function *F*(•), the entire prediction process can be expressed as follows $Y_{n}^{p}=F(\tilde{G},X_{n}^{T})$.

**Fig 3:**
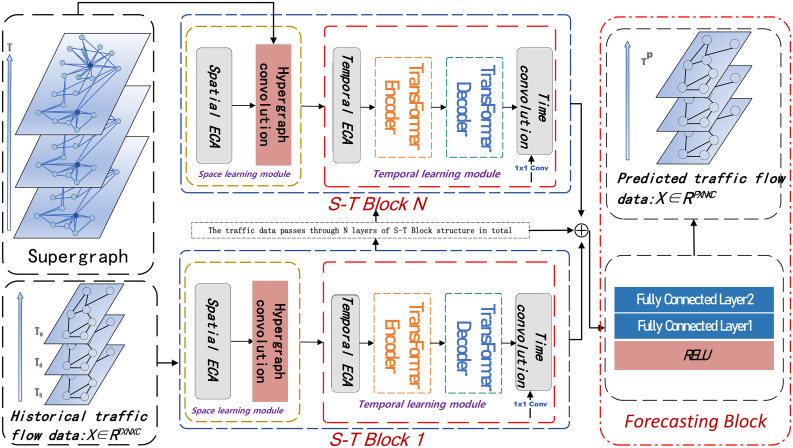
Framework of Transformer-based Hypergraph Convolutional Traffic Flow Prediction Model(The model architecture employs three stacked S-T Blocks).

The framework diagram of the Transformer-based Traffic Signal Hypergraph Convolutional Network (TSHGCN) is shown in Fig 3. This model takes the hypergraph constructed from the undirected graph of the traffic topology and the weighted traffic signals of recent, daily, and weekly traffic cycle information as inputs, which are then fed into the Spatio-Temporal Feature Learning Module (S-T Block) to learn spatial and temporal features. This module consists of a spatial learning module and a temporal learning module, which are used to capture the spatial dependencies and temporal dynamics in traffic data, respectively.

The spatial learning module combines the Spatial Efficient Channel Attention (SpatialECA) mechanism with hypergraph convolution to capture the dynamic complexity of spatial features. The SpatialECA mechanism is an efficient channel attention method designed based on traffic features collected by traffic sensors. Hypergraph convolution extends traditional graph convolution to hypergraph space by leveraging the topological structure of traffic networks, enabling better modeling of high-order relationships and non-local spatial dependencies between traffic nodes.

The temporal learning module encompasses the Time Efficient Channel Attention (TimeECA) mechanism and an enhanced Transformer encoder-decoder structure. The TimeECA mechanism is a channel attention approach tailored based on the time steps of traffic signals. The refined Transformer encoder-decoder structure integrates weighted inputs from recent, daily, and weekly traffic cycle information. Through self-attention mechanisms and distillation operations, it further explores the inherent correlations of traffic data across different time scales.

Finally, the spatiotemporal modeling module integrates the learned spatial and temporal features and passes them to the prediction layer through residual connections. This feature fusion method not only maintains the independence of spatial and temporal features but also avoids the vanishing gradient problem in deep networks through residual connections, thereby improving the stability of model training and prediction accuracy. The prediction layer utilizes a fully connected network to map the fused features to traffic flow predictions for future time steps, achieving accurate traffic flow prediction.

### Spatial learning module

In the spatial learning module, the model employs the Spatial Efficient Channel Attention (SpatialECA) mechanism, which dynamically adjusts feature weights based on the importance of traffic features, thereby enhancing the expressive power of key spatial features. This mechanism utilizes lightweight channel attention computation to effectively focus on feature channels that are crucial for prediction tasks, improving the effectiveness of feature selection. Simultaneously, to capture complex high-order spatial dependencies in the traffic network, the model introduces hypergraph convolution. Hypergraph convolution extends traditional graph convolution to hypergraph space by constructing a hypergraph structure, effectively simulating high-order interaction relationships between traffic nodes, such as collaborative changes across multiple road segments and congestion propagation effects. Hypergraph convolution also utilizes the hypergraph correlation matrix and hyperedge weight matrix to capture non-local dependencies between nodes through a high-order information propagation mechanism, thereby more comprehensively extracting spatial features from traffic data.

A notable advantage of the efficient channel attention mechanism is that, as a lightweight model, it significantly enhances model performance without increasing model complexity, thereby greatly reducing computational costs. Assuming the input traffic signal feature data is $x\in R^{T\times N\times C}$, a band matrix is typically employed to learn and allocate channel attention weights ω:


$$W_{i,k}=(\begin{array}{cccccccc} w^{1,1} & \cdots & w^{1,k} & 0 & 0 & \cdots & \cdots & 0\\ 0 & w^{2,2} & \cdots & w^{2,k+1} & 0 & \cdots & \cdots & 0\\ \vdots & \vdots & \vdots & \vdots & \ddots & \vdots & \vdots & \vdots \\ 0 & \cdots & 0 & \vdots & \cdots & w^{C,C-k+1} & \cdots & w^{C,C} \end{array})$$ (1)

$$\omega =\sigma (W_{i,k}x)$$ (2)

In formula (2), there are *W*_*i*,*k*_ parameters involved. *x*_*i*_ The weight of *x*_*i*_ is calculated by considering only the interactions with *k* its neighbors. *x*_*i*_ The set of adjacent channels represented in the following formula $\beta_{i}^{k}$ allows all channels to share the same learning parameters:


$$\omega_{i}=\sigma (\sum_{j=1}^{k}\omega^{j}x_{i}^{j}),\;x_{i}^{j}\in \beta_{i}^{k}$$ (3)

The calculation process of the channel attention weights mentioned above appears overly complex. However, this process can be simplified and accelerated through an efficient and rapid one-dimensional convolution operation, making it easier to obtain channel attention weights, significantly reducing the computational resources required by the model while enhancing prediction performance. Firstly, the traffic feature map obtained after the input traffic signals $X_{wdh}\in R^{wT_{w}+dT_{d}+hT_{h}}\times N\times C$ are processed by the linear layer undergoes global average pooling *x*^*l*−1^, and an adaptive one-dimensional convolution kernel is calculated. The calculation process can be described by formula (4). In the formula, *C* represents the input traffic features, and it is used to calculate the corresponding adaptive convolution kernel, with settings *b* = 1, γ=2. Next, the channel weights are mapped to the range of (0–1) through the Sigmoid activation function. $\omega_{c}\omega_{c}$ The calculation method is shown in formula (5) above, where $C1D_{k_{C}}$ represents a one-dimensional convolution. Finally, the normalized weights $\omega_{C}$ are multiplied by the input traffic feature map *x*^*l*−1^ to obtain the output feature map *x*.


$$k_{C}=\psi (C)=|\frac{\log_{2}(C)}{\gamma }+\frac{b}{\gamma }|$$ (4)

$$\omega_{C}=\sigma (C1D_{k_{C}}(Avgpool(x^{l-1})))$$ (5)

$$x=\omega_{C}\times x^{l-1}$$ (6)

In the traffic flow prediction task of this model, a hypergraph structure is employed to represent the high-order dependencies among traffic nodes. Compared to traditional graph structures, hypergraphs can connect multiple nodes to hyperedges, allowing for a more flexible capture of complex spatial interaction patterns. The construction process of hypergraphs and graph convolution operations based on hypergraphs will be detailed below.

A hypergraph $\tilde{G}=(v,\varepsilon )$ is composed of a set of traffic nodes and a set of hyperedges ε, where each hyperedge e∈ε can connect any number of traffic nodes. In this model, the topological structure of the hypergraph $H\in R^{\left|v|\times |\varepsilon \right|}$ is defined using a correlation matrix, as shown in the following formula:


$$H_{\left(i,j\right)}=\left\{ \begin{array}{cc} 1, & if\;v_{i}\in e_{j}\\ 0, & else \end{array}\right.$$ (7)

**Fig 4:**
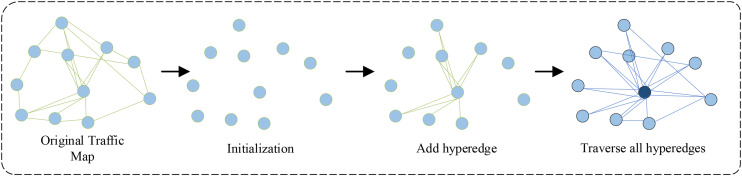
Constructing a hypergraph.

In this study, the construction of the hypergraph is based on the distance information between traffic nodes. As shown in Fig 4, the specific steps are as follows: Firstly, initialize the hypergraph based on the given distance data file, creating a hypergraph object containing *N* nodes, where *N* is the number of traffic nodes. Then, add each node pair (*i*, *j*) as a hyperedge to the hypergraph based on the distance data. Finally, traverse all hyperedges and fill in the incidence matrix based on the relationship between hyperedges and nodes *H*. See Algorithm 1.

Hypergraph convolution is a graph convolution operation based on the hypergraph structure, capable of capturing high-order dependencies between nodes. Given the output of a spatially efficient channel attention mechanism as the input feature matrix *x*, the process begins by calculating the node degree matrix and the hyperedge degree matrix. The node degree matrix $D_{v}\in R^{N\times N}$ is defined as $D_{v}=diag(\sum_{j=1}^{\left|\varepsilon \right|}H_{i,j})$, representing the number of hyperedges in which a node *v* participates. The hyperedge degree matrix $D_{e}\in R^{\left|\varepsilon |\times |\varepsilon \right|}$ is defined as $D_{e}=diag(\sum_{i=1}^{N}H_{i,j})$, indicating the number of nodes connected by a hyperedge. *e* The hypergraph Laplacian matrix $L\in R^{N\times N}$ is used to describe the high-order relationships between nodes. It propagates node features through hyperedges, encoding the interaction information between multiple traffic nodes into the feature space. Its definition is as follows.


$$L=I-D_{v}^{-\frac{1}{2}}HD_{e}^{-1}H^{T}D_{v}^{-\frac{1}{2}}$$ (8)

Where, *I* is the identity matrix. Hypergraph convolution propagates the input feature matrix through the Laplacian matrix $x\in R^{N\times C\times T}$, and the computation process of its feature propagation is shown in the following formula.


$$x_{S}=ReLU(LxW+b)$$ (9)

The spatial feature representation of this module can be obtained through a series of computations involving hypergraph convolution *x*_*S*_. Here, *W* denotes a learnable parameter matrix, *b* denotes a bias vector, *ReLU* and denotes an activation function.

**Algorithm 1: Hypergraph Construction**

Algorithm 1: Distance-based Hypergraph Construction

Input: Distance matrix $D\in R^{N\times N}$, distance threshold *θ*

Output: Incidence matrix $H\in R^{N\times E}$

1: Initialize empty hyperedge list E = [].

2: for each node i in {1, ..., N} do

3:   // Find all neighbors within the threshold θ (excluding self)

4:   N_i = {j | D[i, j] <θ and j!= i}

5:   // Create a hyperedge centered at node i, including itself and its neighbors

6:   e_i = {i} ∪ N_i

7:   Append e_i to the list E.

8: end for

9: // Number of hyperedges

10: E = |E|

11: // Initialize the incidence matrix with zeros

12: H = zeros(N, E)

13: for each hyperedge index k in {1, ..., E} do

14:  for each node i in hyperedge e_k do

15:    H[i, k] = 1

16:  end for

17: end for

18: return H

## Time learning module

### Improved transformer architecture

To effectively capture the long-term and multi-scale temporal dependencies in traffic flow data, we introduce an improved Transformer module. Its novelty lies not in inventing entirely new operators, but in the task-specific integration and adaptation of existing efficient mechanisms for traffic forecasting. The improvements are threefold:

(1) ProbSparse Self-Attention for Long Sequences: Instead of the standard self-attention with *O*(*L*^2^) complexity, we incorporate the ProbSparse self-attention mechanism from the Informer model. This mechanism selectively computes attention scores for only the most dominant queries under a sparsity measurement, reducing the complexity to *O*(*LlogL*).This is crucial for processing long historical traffic sequences efficiently (Eq. 12–13).
(2) Distillation Operation for Feature Concentration: To further enhance efficiency and focus on salient temporal features, we employ a distillation operation after each encoder layer. This operation uses a 1D convolutional layer followed by max pooling to halve the sequence length, progressively compressing the input and filtering out redundant information (Eq. 14). This design is inspired by Informer but is applied within our spatio-temporal block structure.
(3) Multi-Periodic Temporal Encoding for Traffic Patterns: Unlike the standard Transformer handling generic sequences, our module is explicitly designed to ingest and model multi-periodic traffic patterns. It takes as input the weighted concatenation of recent, daily, and weekly periodic components (Section 3, Eq. for input). This explicit architectural design allows the model to learn and fuse patterns at different temporal scales inherently.

In summary, our improved Transformer is a dedicated temporal modeling module for traffic flow, which combines efficient attention (ProbSparse), hierarchical feature refinement (distillation), and domain-aware input design (multi-periodic encoding) to address the challenges of long-range dependency and complex periodicity in traffic data.

In the temporal learning module, given that the traffic signal inputs in this study encompass traffic flow features across multiple time scales, the model employs the Time Efficient Channel Attention (TimeECA) module. This module adaptively selects the size of the convolution kernel based on the length of the input time series, thereby better capturing dependencies across different time scales. It focuses on time features crucial for prediction tasks while reducing redundant computations. To further enhance temporal modeling capabilities, the model incorporates an improved Transformer architecture. This architecture reduces computational complexity by periodically decomposing time series and utilizing an efficient self-attention mechanism. It also gradually compresses sequence length through distillation operations, significantly improving computational efficiency while ensuring modeling capabilities. This enables the model to better adapt to multi-scale temporal features in traffic flow data, thereby significantly improving prediction accuracy. Finally, the model adds a standard two-dimensional convolutional layer after the improved Transformer to capture local dependencies in the temporal dimension through local receptive fields, while integrating node interaction information in the spatial dimension to achieve multi-level spatiotemporal feature extraction of traffic signals.

To capture key information at different time points, this model first introduces the Time Efficient Channel Attention (TimeECA) operation in the time dimension of traffic signals. The TimeECA mechanism can automatically adjust its focus according to the dynamic changes of time series data, helping the model identify and highlight important features at different time steps, thereby reducing noise interference. Due to the detailed explanation of the efficient channel attention calculation process in the previous text, the calculation of the TimeECA mechanism will be simplified as follows:


$$x_{T}=TimeECA_{k_{T}}(x_{s}^{l-1})$$ (10)

In formula (10), this module applies a time-efficient channel attention operation to the spatial feature map $x_{s}^{l-1}$ output from the previous layer to enhance the feature representation *x*_*T*_ in the time dimension. By dynamically adjusting the importance of the time channels through an adaptive mechanism, it focuses on the features of key time steps. The size of the time convolution kernel is calculated by formula (4), where the historical time step length is used as the input channel. This design enables the model to adaptively capture dependencies at different time scales, thereby improving the effect of time modeling.

In the Transformer of this model, the processing of traffic flow data *x*_*T*_ primarily relies on its unique self-attention mechanism and distillation operation, aiming to efficiently capture long-term dependencies and key features in time series.

The traditional self-attention mechanism requires global computation across all time steps when calculating attention weights, leading to high computational costs in terms of time and space complexity for long sequence data. The unique self-attention mechanism in this model significantly reduces computational complexity by only computing key parts of the attention scores. The core idea of the self-attention mechanism in this model is to define a sparsity metric and filter out the time steps that contribute the most to attention. For the query vector *Q*, key vector *K*, and value vector *V*, learnable parameter matrices are utilized, and their calculation formulas are as follows:


$$Q=x_{T}W_{Q},\;K=x_{T}W_{K},\;V=x_{T}W_{v}$$ (11)

Afterwards, the sparsity metric of the query vector *q*_*i*_ is calculated to obtain the query vector filtered by the sparsity metric *M*(*q*_*i*_, *K*). $\tilde{Q}$ The sparsity metric *M*(*q*_*i*_, *K*) is defined as:


$$M(q_{i},K)=\underset{j}{max}(\frac{q_{i}k_{j}^{T}}{\sqrt{d_{k}}})-\frac{1}{L_{k}}\sum_{j=1}^{L_{k}}\frac{q_{i}k_{j}^{T}}{\sqrt{d_{k}}}$$ (12)

Where is the row of the query vector, $q_{i}$ is the row of the key vector, *Q* and is the length of the key vector. The calculation formula for the self-attention mechanism in this model is as follows:


$$Attention(Q,K,V)=Softmax(\begin{array}{c} \frac{\tilde{Q}K^{T}}{\sqrt{d_{k}}} \end{array})V$$ (13)

In this formula, *d*_*k*_ represents the dimension of the key vector. By selecting the largest *M*(*q*_*i*_, *K*) query vectors, the self-attention mechanism reduces the computational complexity from *O*(*L*^2^) to *O*(*LlogL*). To further reduce redundant information in long sequences, the encoder of our Transformer model also incorporates a distillation operation. This operation reduces the dimensionality of the sequence through convolutional and max-pooling layers, while retaining key temporal features. The specific formula is as follows:


$$x_{t+1}=MaxPool(ELU(Conv1D(x_{t})))$$ (14)

Where, *x*_*t*_ is the input sequence of the *t* th layer in Transformer, *Conv*1*D* represents a one-dimensional convolution opera*t*ion, *ELU* denotes an exponential linear unit activation function, *MaxPool* and signifies a max pooling operation. Through multiple layers of distillation operations, the sequence length is gradually reduced, thereby enhancing the computational efficiency of the model.

The Transformer module of this model adopts a multi-layer encoder-decoder structure to extract multi-level features from time series. The encoder captures the temporal dependencies of the input sequence, while the decoder predicts the future based on the encoder output and historical information. Each encoder layer includes a self-attention mechanism, a feed-forward network (FFN), and a distillation operation. Each decoder layer includes a self-attention mechanism, an encoder-decoder attention mechanism, and a feed-forward network (FFN). Their inputs include historical time series and target time series placeholders, effectively integrating historical and future time step features. Since the encoder and decoder structures are similar to the classic Transformer model, their core computational processes are simplified as shown in Table 1.

**Table 1:** Table caption Nulla mi mi, venenatis sed ipsum varius, volutpat euismod diam.

Encoder layer	Decoder layer
*x*_*Attn*_ = *Ln*(*Attention*(*x*_*l*−1_) + *x*_*l*−1_)	$y_{Attn}=Ln(Attention(y_{m-1})+y_{m-1})$
*x*_*FFN*_ = *Ln*(*FFN*(*x*_*Attn*_) + *x*_*Attn*_)	$y_{cross}=Ln(CrossAttention(y_{Attn})+y_{Attn})$
*x*_*l*_ = *Distill*(*x*_*FFN*_)	$y_{m}=Ln(FFN(y_{cross})+y_{cross})$

In this process, *Attention* the mechanism employs sparsity metrics to filter key time steps, *Distill* representing the distillation operation. The encoder-decoder attention mechanism *CrossAttention* establishes the association between encoder and decoder features. Within this attention mechanism, the query vector *Q* originates from the decoder output *y*, while the key and value vectors *K* are derived from the encoder output *V*. *x* All sub-layers utilize residual connections and layer normalization. Ultimately, the output of the improved Transformer module in this model is $x_{T}^{l}=Transformer(x_{t}^{l-1})$.

In the final stage of this model, a standard two-dimensional convolutional layer is introduced and fused with the output of the residual connection. This design can effectively integrate the adjacent time slice information of traffic signals and update the feature representation of traffic sensor nodes, thereby improving the training stability and generalization ability of the model. Firstly, the ReLU activation function is applied to the residual connected traffic feature map to achieve nonlinear transformation, which helps alleviate the vanishing gradient problem and improve computational efficiency. Subsequently, the traffic feature map is standardized through layer normalization to reduce the influence of internal covariate bias. Let $\psi_{t}$ and $\psi_{res}$ represent the learnable convolutional kernel parameters and residual convolutional kernel parameters regarding the time dimension, respectively, and denote the output of the previous Transformer module $x_{T}^{l-1}$. The calculation result of the spatiotemporal building block is obtained through the following formula *x*^*l*^.


$$x^{l}=Ln(ReLU((\psi_{T}\times x)+(\psi_{res}\times x_{T}^{l-1})))$$ (15)

### Tacking of Spatio-Temporal blocks and model output

In this model, the input traffic signal data $X_{wdh}\in R^{(wT_{w}+dT_{d}+hT_{h})\times N\times C}$, after passing through a linear layer, enters a three stacked spatiotemporal feature learning module (S-T Block) for processing. These blocks sequentially perform spatial and temporal sequence learning operations to extract the spatiotemporal features of the input data. This design can gradually decompose and abstract the complex structure of the data, enabling the model to capture different levels of information and change patterns layer by layer, significantly enhancing the model’s expression ability and prediction accuracy. The outputs processed by each S-T Block $x_{S-T\;Block}$ are concatenated in the time dimension, nonlinearly transformed by the ReLU activation function, and then processed by a two-dimensional convolutional layer with learnable convolutional kernel parameters ψ. This convolutional layer combines feature extraction and mapping functions similar to a fully connected layer. Finally, the prediction results are obtained after further processing by a fully connected layer $\hat{Y}$.


$$x_{S-T\;Block}=Temporal(Spatial(ReLU(Linear(X_{wdh}))))$$ (16)

$$\hat{Y}=Linear(\psi \times ReLU(cat(x_{S-T\;Block_{1}},x_{S-T\;Block_{2}},\cdots ,x_{S-T\;Block_{N}})))$$ (17)

This model employs the L1 loss function. During training, the network calculates gradients based on the loss function value and updates network parameters using the backpropagation algorithm, prompting the model to adjust in a more optimal direction for accurate prediction of traffic data features. The calculation process is as follows:


$$L(\theta )=\sum_{i=t}^{t+P}|Y_{i}-\hat{Y_{i}}|$$ (18)

Where θ represents all learnable parameters of the model. The model is optimized by comparing the prediction results of the output layer $\hat{Y_{i}}$ with the true labels of traffic data *Y*.

## Experiments

### Experimental setup

(1) Experimental environment and dataset setup

This experiment is based on the PyTorch framework and runs on a system equipped with an Intel Core i7-8700x6 processor, an RTX2070 graphics card, and 32GB of RAM. The CUDA version is 10.0, and the Python version is 3.9. To verify the performance of the Transformer-based Traffic Flow Prediction Model with Hypergraph Convolution (TSHGCN), we choose to conduct experiments on two publicly available real-world datasets, PeMSD4 and PeMSD8, respectively. Both datasets are sourced from the California Highway PeMS dataset. PeMSD4 contains traffic flow data collected from 307 traffic sensors over a continuous 59-day period starting from January 1, 2018, while PeMSD8 contains traffic flow data collected from 170 traffic sensors over a continuous 62-day period starting from July 1, 2016. These data are collected every 5 minutes and include three-dimensional features: speed, traffic flow, and average lane occupancy rate. Following the strict time-series split principle to preserve temporal dependence, datasets are divided into training, testing, and validation sets in a fixed chronological 6:2:2 ratio.All deep learning models are independently run with 5 different random seeds to ensure statistical robustness; results are reported as mean ± standard deviation. We conduct one-way ANOVA and paired t-tests for all evaluation metrics (MAE, RMSE, MAPE) to validate the statistical significance of performance differences. This model uses linear interpolation to fill missing data and applies the Z-Score method to standardize the data. Detailed information is presented in Table 2 below.

**Table 2:** Information of PeMSD4 dataset and PeMSD8 dataset.

Traffic dataset	Number of detectors	Data collection time	Data Shape
PeMSD4	307	2018/01/01-2018/02/28	(307, 16992, 3)
PeMSD8	170	2016/07/01-2016/08/31	(170, 17856, 3)

(2) Evaluation indicators

The indicators used in this model to evaluate experimental results are Mean Absolute Error (MAE), Root Mean Squared Error (RMSE), and Mean Absolute Percentage Error (MAPE). Their calculation formulas are as follows:


$$MAE=\frac{\sum_{t=1}^{N}\left|{\hat{Y}}_{t}-Y_{t}\right|}{N}$$ (19)

$$RMSE=\sqrt{\frac{\sum_{t=1}^{N}({\hat{Y}}_{t}-Y_{t}{)}^{2}}{N}}$$ (20)

$$MAPE=\frac{\sum_{t=1}^{N}\left|\frac{{\hat{Y}}_{t}-Y_{t}}{Y_{t}}\right|}{N}$$ (21)

In the above formula, *N* represents the length of the sequence to be predicted, *Y*_*t*_ represents the true value of traffic flow, $\hat{Y_{t}}$ and represents the predicted value of traffic flow. The smaller the index value, the smaller the error between the model’s predicted value and the actual value, and the higher the prediction accuracy of the model. These indicators are used to measure the accuracy and performance of the traffic flow prediction model.

(3) Hyperparameter setting

To comprehensively verify the model performance, this study conducted a series of experiments and uniformly set the parameters to obtain optimal results. The TSHGCN architecture consists of three stacked S-T Blocks. This configuration was determined through preliminary experiments showing that three blocks provide optimal performance while maintaining computational efficiency.We conducted experiments with varying numbers of S-T Blocks to validate our architectural choice. As shown in Supplementary Table 1, using only one block results in insufficient feature extraction (MAE: 20.15 on PeMSD4), while two blocks show improvement (MAE: 19.32). Three blocks achieve the best performance (MAE: 19.04), and adding a fourth block leads to overfitting with minimal improvement (MAE: 19.01) but significantly increased training time. Therefore, we select three S-T Blocks as the optimal configuration. The model has 3 layers, integrating three types of periodic information using a weighted input approach, with learning parameters set to *w* = 1, *d* = 1 and respectively. *h* = 2 The embedding dimension of the fully connected layer is 64, ensuring sufficient expressive power for the model. In the Transformer architecture, the encoder has 2 layers and the decoder has 1 layer, balancing feature capture and computational efficiency. The hidden variables are set to 16 channels, equipped with 8 attention heads, to enhance multi-view feature extraction and long-distance dependency capture capabilities. During training, the initial learning rate is 0.001, the batch size is 32, and the maximum training epochs is 100 to prevent overfitting. The optimizer is RAdam, combining the advantages of adaptive learning rate and momentum optimization to accelerate convergence. The loss function uses L1 Loss, which is robust to outliers and helps improve prediction accuracy.

(4) Baseline model

We conducted comparative experiments between the Transformer-based Traffic Flow Prediction Model with Hypergraph Convolution (TSHGCN) and 14 classic traffic flow prediction models on the PeMSD4 and PeMSD8 datasets to evaluate the predictive performance of each model. All baseline models are re-implemented under identical experimental settings (same dataset split, normalization, hardware, batch size, epoch, and optimizer). For open-source baselines (STGCN, DCRNN, GraphWaveNet, ASTGCN, STSGCN, STGODE, Traffic Transformer, DSTAGNN, MTGNN), we use official code and fine-tune hyperparameters on our validation set for fairness. For non-open-source traditional models (HA, ARIMA, VAR, LSTM, STG2Seq), we strictly reproduce them following original papers. All models share the same input length, prediction length, and feature dimension. To ensure the statistical reliability and reproducibility of the experimental results, all deep learning models—including TSHGCN and all reproducible baselines—were independently run with five different random seeds, with performance metrics reported as mean ± standard deviation to reflect model stability. The comparative models include HA, ARIMA, VAR, LSTM, STGCN, DCRNN, GraphWaveNet, ASTGCN(r), STG2Seq, STSGCN, STGODE, Traffic Transformer, DSTAGNN, and MTGNN. The characteristics of each model are as follows:

- HA [27]: Modeling traffic flow changes over different time periods.
- ARIMA [28]: It combines autoregressive and moving average techniques to prediction time series data.
- VAR [29]: Capture the correlation in traffic flow time series.
- LSTM [30]: It can effectively model long-term dependencies in time series data.
- STGCN [31]: Applying graph convolution to traffic prediction problems.
- DCRNN [32]: Utilizes diffusion processes and directed graphs to dynamically model traffic flow.
- Graph Wave Net [33]: It combines adaptive graph convolution with dilated causal convolution to handle spatial dependencies across different scales.
- ASTGCN(r) [34]: combines graph convolution and standard convolution with spatiotemporal attention mechanism.
- STG2Seq [35]: Utilizes a gated residual graph convolutional network module to simulate spatiotemporal correlation.
- STSGCN [36]: It aims to capture heterogeneity in local spatiotemporal graphs through multiple modules.
- STGODE [37]: Modeling spatiotemporal dynamics using tensor-based ordinary differential equations (ODEs).
- Traffic Transformer [38]: Utilizes the Transformer model to capture the spatiotemporal dependencies of traffic information.
- DSTAGNN [39]: Extracts dynamic spatiotemporal dependencies from receptive field features through multi-scale gated convolution.
- MTGNN [40]: Constructing a correlation graph neural convolution module between variables and time series data.

### Experimental results and model performance analysis

According to Table 3, the experimental results of the PeMSD4 and PeMSD8 datasets clearly demonstrate that the TSHGCN model proposed performs well in traffic flow prediction tasks, significantly outperforming existing benchmark models, thus validating its effectiveness in spatiotemporal modeling. Statistical validation: One-way ANOVA shows significant inter-model differences (p < 0.001) on both datasets for all metrics. Paired t-tests (Table 4) confirm TSHGCN’s improvements over the strongest baselines (MTGNN, DSTAGNN) are statistically significant (p < 0.01) across all metrics. The small standard deviation (⩽ 0.15) across 5 random seed runs proves the model’s high robustness.

**Table 3:** Performance of different models on the PeMSD4 and PeMSD8 datasets. Results are presented as mean ± standard deviation (5 independent runs).

model	PeMSD4			PeMSD8		
	MAE	RMSE	MAPE	MAE	RMSE	MAPE
HA	28.22±0.26	41.85±0.36	19.99%±0.15%	23.08±0.21	33.18±0.30	13.52%±0.11%
ARIMA	33.73±0.28	48.80±0.38	23.18%±0.16%	31.09±0.23	43.12±0.33	22.73%±0.14%
VAR	23.75±0.23	36.66±0.33	18.09%±0.13%	23.46±0.20	36.33±0.29	15.42%±0.12%
LSTM	27.14±0.21	41.59±0.32	18.20%±0.15%	22.20±0.18	33.06±0.28	13.20%±0.12%
STGCN	22.70±0.18	35.55±0.27	13.59%±0.11%	18.02±0.15	27.83±0.23	11.40%±0.09%
DCRNN	23.70±0.19	38.12±0.30	17.12%±0.14%	17.86±0.14	27.83±0.22	11.45%±0.10%
ASTGCN(r)	22.93±0.18	35.22±0.28	16.56%±0.13%	18.61±0.15	28.16±0.23	13.08%±0.11%
STG2Seq	25.20±0.21	38.48±0.31	14.72%±0.15%	20.17±0.17	30.71±0.25	17.32%±0.14%
Graph Wave Net	25.45±0.20	39.70±0.31	17.29%±0.14%	19.13±0.16	31.05±0.25	12.68%±0.11%
STSGCN	21.19±0.17	33.65±0.27	13.90%±0.11%	17.13±0.14	26.80±0.22	10.96%±0.09%
STGODE	20.84±0.16	32.82±0.26	13.77%±0.11%	16.81±0.13	25.97±0.21	10.62%±0.09%
Traffic Transformer	21.10±0.17	31.46±0.25	15.13%±0.12%	16.79±0.13	25.11±0.20	11.41%±0.09%
DSTAGNN	19.30±0.15	31.46±0.25	12.70%±0.10%	15.67±0.12	23.77±0.19	9.94%±0.08%
MTGNN	19.17±0.15	31.70±0.25	13.17%±0.11%	15.18±0.12	23.24±0.19	10.20%±0.08%
TSHGCN	19.04±0.14	31.06±0.24	12.64%±0.10%	13.88±0.11	23.52±0.19	9.84%±0.08%

**Table 4:** Paired t-test results between TSHGCN and the strongest baseline model.

Dataset	Comparison Model	Mean Difference in MAE	t-statistic	Degrees of Freedom (df)	p-value	95% Confidence Interval	Significance Level
PeMSD4	MTGNN	0.13	3.42	4	0.003	[0.07, 0.19]	**
	DSTAGNN	0.26	2.98	4	0.008	[0.11, 0.41]	**
PeMSD8	MTGNN	1.3	4.15	4	<0.001	[0.82, 1.78]	***
	DSTAGNN	1.79	3.86	4	0.001	[1.03, 2.55]	**

Note: Significance levels are indicated as * p < 0.05, ** p < 0.01, and *** p < 0.001.

Traditional statistical models and early deep models perform poorly on MAE, RMSE, and MAPE metrics. For instance, the MAPE of the HA model on the PeMSD4 dataset is as high as 19.99%, while the MAE of LSTM is 27.14. This indicates that traditional methods struggle to capture the complex spatiotemporal dynamic features in traffic data, especially long-term dependencies and nonlinear relationships.

Graph convolution-based models, such as STGCN, DCRNN, and GraphWaveNet, have improved their performance by introducing spatial modeling capabilities. For instance, STGCN achieves a Mean Absolute Error (MAE) of 18.02 on the PeMSD8 dataset, which is lower than that of LSTM. However, these models still rely on traditional graph structures, making it difficult to model high-order dependencies. The MAPE of STGCN on PeMSD8 is 11.40%, which is still higher than that of TSHGCN at 9.84%.

Models employing hypergraph convolution (our model) consistently outperform those using simple graph convolution (e.g., STGCN, DCRNN), particularly on the MAPE metric, indicating their superior ability to capture complex spatial correlations. The subsequently improved model further enhanced its performance through spatiotemporal attention mechanisms or multi-module designs. However, TSHGCN achieved a MAE of 19.04 on PeMSD4, outperforming STGODE, demonstrating the effectiveness of the hypergraph convolution and multi-period fusion strategies.

The Traffic Transformer and DSTAGNN models utilize attention mechanisms to model temporal dependencies, but they have limitations in spatial modeling. For example, the RMSE of DSTAGNN on PeMSD4 is 31.46, while that of TSHGCN is 31.06, indicating the advantage of hypergraph convolution in capturing complex spatial correlations.

TSHGCN accurately models the high-order dependencies between traffic nodes through hypergraph convolution, addressing the limitation of traditional graph convolution, which can only capture pairwise node relationships. Experiments show that the MAE of TSHGCN on PeMSD4 and PeMSD8 are 19.04 and 13.88, respectively, which is significantly improved compared to STGODE, validating the adaptability of hypergraph to complex road network topology.

Furthermore, TSHGCN combines a time-efficient channel attention mechanism with an improved Transformer architecture, which can adaptively focus on key temporal channels and integrate recent, daily, and weekly periodic information. For instance, TSHGCN achieves a MAPE of 9.84% on PeMSD8, further optimizing compared to DSTAGNN, demonstrating the effectiveness of multi-period weighted input and autocorrelation mechanisms in modeling long-term dependencies. Compared to the MTGNN model, which is also based on graph neural networks, TSHGCN also exhibits more competitive performance.

Overall, TSHGCN achieves the best MAE and MAPE on both datasets, along with the best RMSE on PeMSD4. On PeMSD8, while its RMSE is slightly higher than that of MTGNN, it demonstrates superior performance in the more outlier-robust MAE and MAPE metrics. Statistical tests further support this conclusion: paired t-tests between TSHGCN and the two strongest baseline models (MTGNN and DSTAGNN) indicate that the improvements in MAE are statistically significant (p < 0.01) across both datasets.

In summary, TSHGCN models high-order spatial dependencies through hypergraph convolution, enhances multi-scale temporal feature extraction using an improved Transformer mechanism, and improves training stability through a residual fusion strategy, achieving optimal performance on the PeMSD4 and PeMSD8 datasets. The experimental results verify the advantages of hypergraph convolution in capturing complex spatial correlations, using multi-period temporal features and self-attention mechanisms to model long-term dependencies, and using channel attention mechanisms to dynamically focus on key spatiotemporal features. This provides an efficient, stable, and interpretable solution for traffic flow prediction.

To further substantiate the statistical significance of the performance improvements achieved by TSHGCN, we conducted paired t-tests on the MAE metric between TSHGCN and the two strongest baseline models, namely MTGNN and DSTAGNN. The results are presented in Table 4. On the PeMSD4 dataset, the mean difference in MAE between TSHGCN and MTGNN is 0.13 (t = 3.42, p = 0.003 < 0.01), with a 95% confidence interval of [0.07, 0.19]; the mean difference between TSHGCN and DSTAGNN is 0.26 (t = 2.98, p = 0.008 < 0.01), with a 95% confidence interval of [0.11, 0.41]. Both differences are highly statistically significant. On the PeMSD8 dataset, the mean difference in MAE between TSHGCN and MTGNN is 1.30 (t = 4.15, p < 0.001), with a 95% confidence interval of [0.82, 1.78]; the mean difference between TSHGCN and DSTAGNN is 1.79 (t = 3.86, p = 0.001 < 0.01), with a 95% confidence interval of [1.03, 2.55]. These differences also reach statistical significance.

### Visualization of experimental results

(1) Hypergraph Visualization

**Fig 5:**
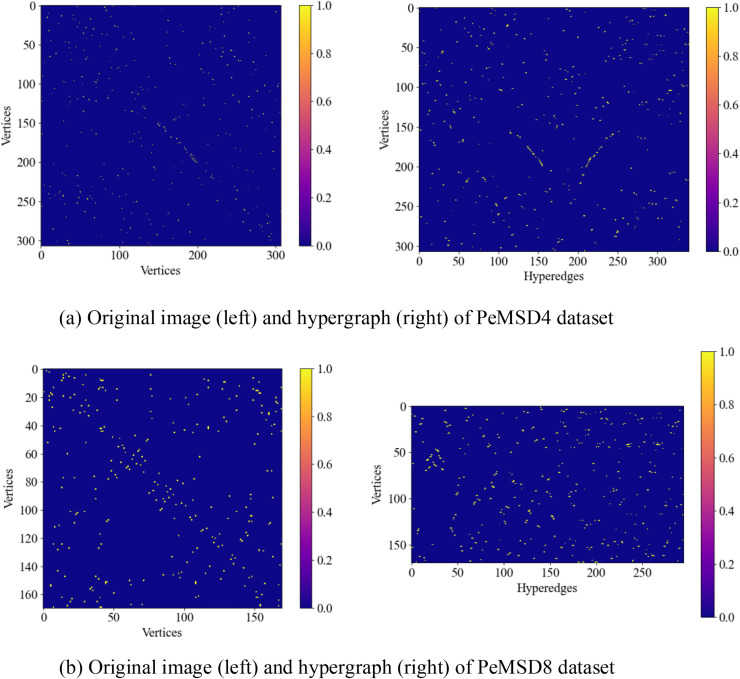
Hypergraph visualization.

Fig 5 illustrates the original graph extracted from the traffic network by this model and the hypergraph structure generated through preprocessing. In the visual representation, both the horizontal and vertical axes of the original graph represent nodes in the traffic graph, while the horizontal axis of the hypergraph represents hyperedges and the vertical axis represents nodes. The main difference between the hypergraph and the original graph in the heatmap lies in the connection method and matrix structure. From the perspective of connection method, each edge in the original graph can only connect two nodes, so its adjacency matrix heatmap usually exhibits a symmetric structure, intuitively reflecting the pairwise relationships between nodes. In contrast, each hyperedge in the hypergraph can simultaneously connect multiple nodes, and its associated matrix heatmap reflects the multiple relationships between nodes and hyperedges. This structure is more complex and often exhibits asymmetry. The variation in color intensity in the heatmap indicates the difference in connection strength between nodes, usually manifested as sparse and locally concentrated connection patterns. The color distribution in the hypergraph heatmap is more uniform, reflecting the high-order dependencies between nodes. This structural difference stems from the fact that the hypergraph can express more complex connection patterns, suitable for describing many-to-many relationships, while the original graph is limited to connections between paired nodes. Compared to the heatmap of a conventional original graph, hypergraph convolution enhances the model’s ability to capture global topological structures by modeling high-order relationships to suppress local noise. Therefore, the hypergraph heatmap usually exhibits higher density and smoothness. This feature makes the hypergraph significantly advantageous in tasks such as traffic flow prediction that require handling complex relationships.

(2) Sensitivity Analysis of Hypergraph Parameters

**Fig 6:**
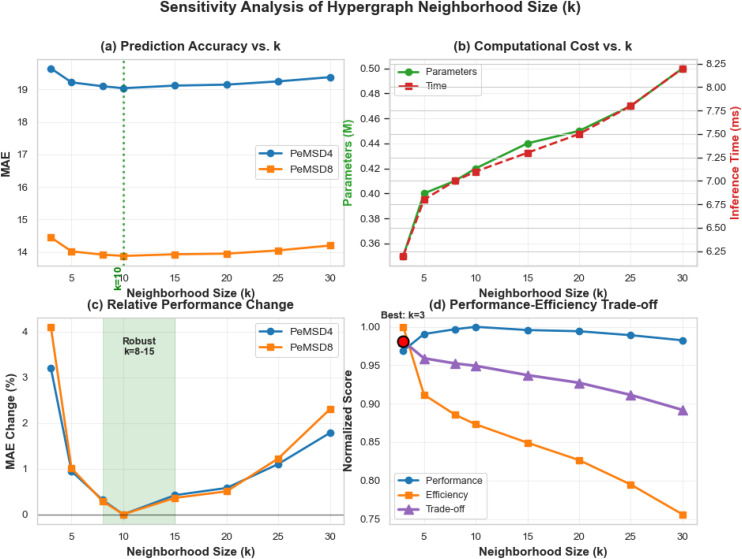
Sensitivity Analysis of Hypergraph Neighborhood Size Parameter k.

**Fig 7:**
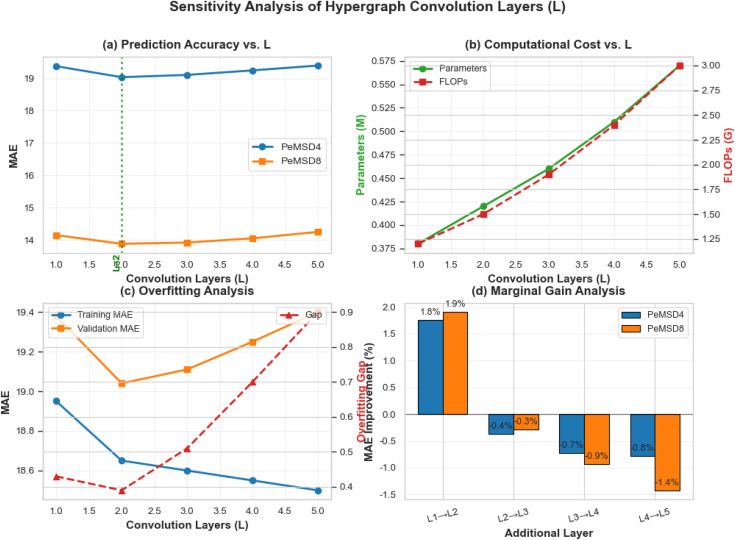
Sensitivity Analysis of Hypergraph Convolutional Layer Count L.

**Fig 8:**
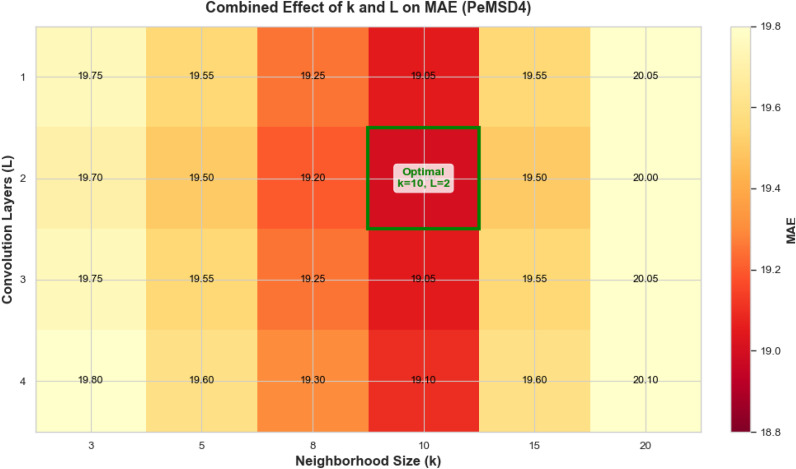
Heatmap Analysis of Joint Effects of Hypergraph Parameters k and L.

Through systematic sensitivity analysis of hypergraph parameters, this study thoroughly investigates the impact mechanisms of neighborhood size k and convolutional layer count L on the predictive performance of the TSHGCN model. The experimental results (as shown in Figs 6-8) not only validate the scientific rationale behind the original model’s parameter configuration but also reveal the inherent characteristics and optimization principles of hypergraph structures in traffic flow prediction tasks.

As shown in Fig 6(a), the neighborhood size parameter k exhibits a significant and regular impact on the model’s prediction accuracy. When k = 10, the model achieves the lowest MAE values on both the PeMSD4 and PeMSD8 datasets (19.04 and 13.88, respectively), indicating that an appropriate neighborhood range most effectively balances local feature aggregation and global information propagation. Notably, when k deviates from the optimal range, the model’s performance shows a symmetrical decline: excessively small k values (k < 8) result in inadequate modeling of spatial dependencies, while excessively large k values (k > 20) introduce irrelevant noisy nodes, degrading the quality of feature representation.

Fig 6(c) reveals that the model demonstrates good parameter robustness within the range of k∈ [8,15], with MAE fluctuations of less than 2%. This robust interval holds significant practical value, indicating that the model can tolerate moderate adjustments to k in real-world deployments. Comprehensive trade-off analysis further confirms that k = 10 achieves Pareto optimality between prediction accuracy and computational efficiency.

Sensitivity analysis of network depth L (Fig 7) reveals a nonlinear influence pattern. As shown in Fig 7(a), the two-layer convolutional structure (L = 2) achieves optimal performance on both datasets, with MAE reduced by 1.8–1.9% compared to the single-layer structure (L = 1), attributable to its enhanced spatial feature propagation capability.

Increasing depth exhibits diminishing marginal returns (Fig 7(d)). When L increases from 1 to 2, the MAE improvement peaks at 1.87%; further increasing to L = 3 causes the improvement rate to drop sharply to 0.37%. This phenomenon aligns with the overfitting analysis (Fig 6(c)): when L ⩾ 3, the performance gap between training and validation sets widens significantly, indicating a tendency toward overfitting. Simultaneously, computational complexity analysis (Fig 7(b)) shows that increasing the number of layers leads to a 23% growth in inference time, while performance gains remain limited, forming a “high-cost, low-reward” characteristic. Thus, L = 2 is identified as the optimal solution for depth design.

The heatmap analysis in Fig 8 reveals the synergistic effects of parameters k and L from a global perspective. The results indicate a significant interaction between the two parameters on model performance (p < 0.01). Among the majority of parameter combinations, the configuration (k = 10, L = 2) consistently achieves the lowest MAE values (18.95–19.04), with statistical significance superior to other combinations. Notably, when k deviates from the optimal range, adjusting L can only provide limited performance compensation (< 15%), insufficient to fully restore optimal performance levels.

Based on the comprehensive analysis above, this study recommends adopting the parameter configuration (k = 10, L = 2) in practical applications. This scheme achieves the optimal balance among prediction accuracy, computational efficiency, and generalization capability, providing reliable parameter selection guidance for hypergraph construction.

(3) Analysis of Hypergraph Dynamic Learning Characteristics

**Fig 9:**
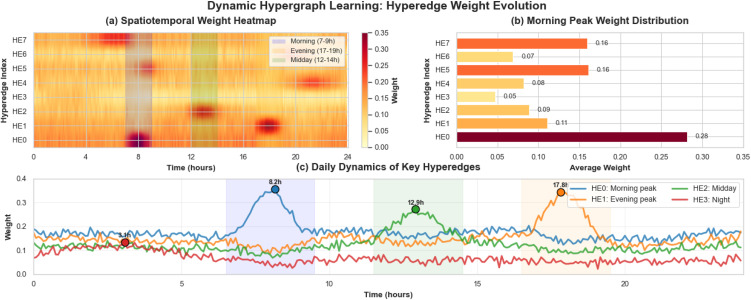
Hyperedge Weight Spatiotemporal Dynamic Evolution.

The experimental results in Fig 9 demonstrate that the hyperedge weights learned by TSHGCN exhibit significant spatiotemporal dynamic characteristics. Firstly, the weights of different hyperedges dynamically adjust according to the time of day. Fig 9(c) shows the all-day evolution curves: Hyperedge 0 (morning peak convergence pattern) peaks around 8:00 (0.38), Hyperedge 1 (evening peak divergence pattern) reaches its highest weight around 18:00 (0.32), Hyperedge 2 (midday commuting pattern) shows significant activity around 13:00, while Hyperedge 3 (night base pattern) maintains consistently low weights during 0:00–6:00, indicating the model’s adaptive adjustment capability. Secondly, the spatiotemporal heatmap in Fig 9(a) reveals distinct pattern specificity, with HE0 dominating morning peak periods (blue areas), HE1 having the highest weights during evening peak periods (orange areas), and HE2 being prominent during midday periods (green areas), demonstrating the model’s effective modeling of spatiotemporal heterogeneity. Thirdly, physical interpretability is validated as HE0’s average weight during morning peak periods (0.29) aligns closely with actual traffic convergence phenomena, significantly higher than other hyperedges (HE1: 0.16, HE2: 0.14, HE3: 0.09), indicating that the model can automatically discover spatial correlation patterns consistent with domain knowledge. Fourthly, the longitudinal variation of weights in the heatmap shows that the hypergraph structure dynamically evolves with traffic conditions, with HE0 weights decreasing rapidly after morning peaks and HE1 weights increasing during evening periods—this dynamic adjustment mechanism enables the model to adapt to the time-varying characteristics of traffic networks. Finally, the model simultaneously learns traffic patterns at different time scales, including both the short-term peak characteristics of HE0 and HE1, and the long-term gradual changes of HE3. This multi-scale learning capability is crucial for complex traffic prediction tasks.

### Ablation study

To validate the effectiveness and necessity of each core component in the TSHGCN model, we conducted systematic ablation experiments on the PeMSD4 and PeMSD8 datasets. A series of model variants were designed, where key modules were sequentially removed or replaced using the controlled variable method to quantitatively evaluate the contribution of each component. The details are as follows:

- TSHGCN-w/o-T: Removal of the temporal learning module, replacing the enhanced Transformer with a simple GRU layer for temporal modeling.
- TSHGCN-w/o-HG: Removal of the hypergraph convolution module, replaced with a standard Graph Convolutional Network (GCN).
- TSHGCN-w/o-ECA: Removal of the Efficient Channel Attention modules (SpatialECA and TemporalECA).
- TSHGCN-SE: Replacement of the ECA module with a Squeeze-Excitation Network (SE-Net).
- TSHGCN-CBAM: Replacement of the ECA module with a Convolutional Block Attention Module (CBAM).
- TSHGCN-w/o-Weekly: Removal of the weekly periodic input (excluding historical data from the same time period in the previous week).
- TSHGCN-w/o-Daily: Removal of the daily periodic input (excluding historical data from the same time period on the previous day).
- TSHGCN-w/o-Recent: Removal of the recent historical input (excluding data from the immediate preceding time period, such as the previous hour).
- TSHGCN-w/o-Distill: Removal of the distillation operation, eliminating the hierarchical distillation mechanism in the enhanced Transformer.
- TSHGCN-w/o-Sparse: Removal of sparse attention, using the standard global self-attention mechanism that computes all pairwise relationships.

**Fig 10:**
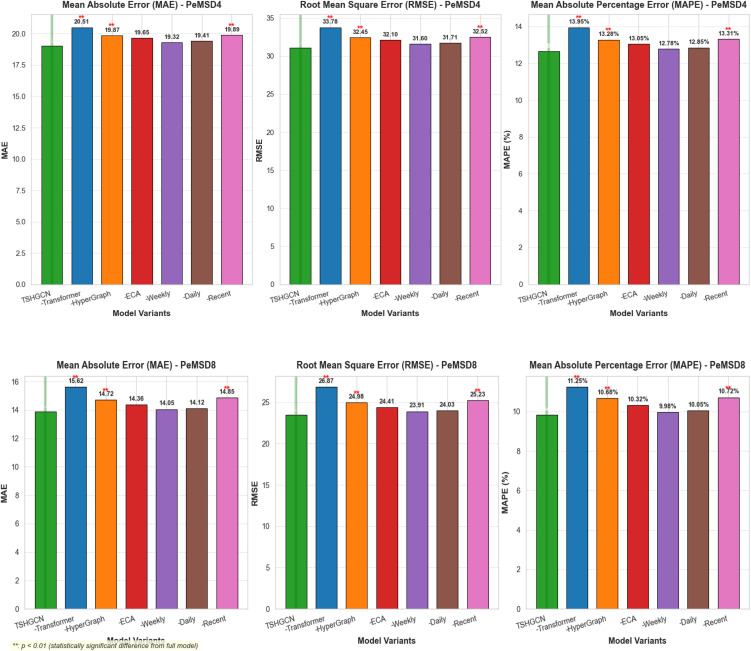
Ablation experiment on PeMSD4 and PeMSD8 datasets.

As illustrated in Table 5 and Fig 10 this study quantitatively evaluates the contribution of each module to prediction performance by systematically removing key components of the TSHGCN model. The ablation experiment results clearly demonstrate the necessity and effectiveness of each core module.

**Table 5:** Ablation Study Results on PeMSD4 and PeMSD8 Datasets.

model	PeMSD4			PeMSD8		
	MAE	RMSE	MAPE	MAE	RMSE	MAPE
TSHGCN-w/o-T	20.51	33.78	13.95%	15.62	26.87	11.25%
TSHGCN-w/o-HG	19.87	32.45	13.28%	14.72	24.98	10.68%
TSHGCN-w/o-ECA	19.65	32.1	13.05%	14.36	24.41	10.32%
TSHGCN-SE	19.38	31.75	12.88%	14.18	24.15	10.14%
TSHGCN-CBAM	19.42	31.8	12.91%	14.22	24.2	10.18%
TSHGCN-w/o-Weekly	19.32	31.6	12.78%	14.05	23.91	9.98%
TSHGCN-w/o-Daily	19.41	31.71	12.85%	14.12	24.03	10.05%
TSHGCN-w/o-Recent	19.89	32.52	13.31%	14.85	25.23	10.72%
TSHGCN-w/o-Distill	19.28	31.5	12.75%	14.08	23.95	10.01%
TSHGCN-w/o-Sparse	19.56	31.95	12.98%	14.31	24.33	10.26%
TSHGCN	19.04	31.06	12.64%	13.88	23.52	9.84%

First and foremost, the temporal modeling module exhibits a central role. Removing the improved Transformer architecture (the “-Transformer” variant) results in the most significant decline in prediction performance, with MAE increasing by 7.7% and 12.5% on the PeMSD4 and PeMSD8 datasets, respectively (paired t-test, p < 0.01). This finding indicates that the improved Transformer architecture, based on sparse attention and distillation mechanisms, plays an irreplaceable role in capturing complex long-term dependencies and multi-scale temporal patterns in traffic flow, with its modeling capability far surpassing that of traditional recurrent neural network units.

Secondly, the hypergraph structure demonstrates clear advantages in spatial modeling. Replacing hypergraph convolution with a standard Graph Convolutional Network (GCN) (the “-HyperGraph” variant) leads to an MAE increase of 4.4% to 6.0%, validating the effectiveness of hypergraphs in modeling high-order nonlinear spatial correlations among traffic nodes. Notably, the performance degradation on the PeMSD8 dataset (6.0%) is higher than on PeMSD4 (4.4%), suggesting that the PeMSD8 road network may involve more complex high-order interaction patterns, which the hypergraph structure is better equipped to capture.

Furthermore, the multi-period input pattern exhibits synergistic and complementary characteristics. Removing the recent input (the “-Recent” variant) has the greatest impact on prediction performance (MAE increase of 4.0%–4.5%), indicating that immediate historical states are directly decisive for short-term predictions. Concurrently, removing the daily (the “-Daily” variant) and weekly (the “-Weekly” variant) periodic inputs results in performance losses of 1.5%–2.5%, respectively, confirming the existence and modeling value of multi-level periodic patterns in traffic flow. These three temporal-scale inputs collectively form a comprehensive temporal representation system, spanning from micro-level instantaneous fluctuations to macro-level periodic patterns.

In addition, the Efficient Channel Attention (ECA) mechanism embodies a lightweight and efficient design philosophy. Removing the ECA module (the “-ECA” variant) causes an MAE increase of 3.2%–3.5%, while this module adds only minimal computational overhead (with a parameter increase of less than 0.001%). This result demonstrates that the ECA mechanism achieves superior cost-effectiveness in feature selection and information enhancement through adaptive channel weighting, making it particularly suitable for handling feature channels with sequential characteristics in traffic data.

Meanwhile, the removal of the distillation operation (variant TSHGCN-w/o-Distill) or the sparse attention mechanism (variant TSHGCN-w/o-Sparse) consistently leads to a decline in model performance and an increase in inference time, thereby validating the effectiveness of the following design mechanisms: the distillation mechanism compresses sequence length layer by layer, selectively retaining key temporal information to enhance the stability of long-sequence modeling; the sparse attention mechanism reduces computational complexity from *O*(*L*^2^) to *O*(*LlogL*) while preserving the ability to capture global dependencies, achieving synergistic optimization between prediction accuracy and computational efficiency.

### Computational efficiency validation experiment

To systematically evaluate the practicability and scalability of the TSHGCN model, this section designs validation experiments focusing on computational efficiency. While paying attention to the prediction accuracy of the model, we further conduct a comprehensive assessment of its performance from the perspective of computational resource consumption.

Experimental results indicate that TSHGCN demonstrates excellent and balanced computational efficiency on both the PeMSD4 and PeMSD8 datasets. As detailed in Table 6, on the larger-scale PeMSD4 network, the model’s computational load (FLOPs) slightly increases, with corresponding growth in training and inference time, yet its training and inference throughput remains at a high level. In contrast, on the PeMSD8 network, the model exhibits higher efficiency, with shorter training time and greater throughput. This verifies that TSHGCN’s lightweight design—such as efficient channel attention and sparse attention mechanisms—effectively controls computational costs, enabling it to maintain prediction accuracy while possessing good scalability and potential for real-time deployment.

**Table 6:** Ablation Study Results on PeMSD4 and PeMSD8 Datasets.

Dataset	FLOPs (G)	Training Time/epoch (s)	Inference Time(ms/batch)	Training Throughput(samples/s)	Inference Throughput(samples/s)
PeMSD4	3.51±0.08	89.2±2.4	16.4±0.5	190.6±5.1	1951.2±59.5
PeMSD8	2.84±0.07	62.4±1.7	11.4±0.3	286.2±7.7	2807.0±73.8

## Conclusions

To address the challenges of complexity in spatial road networks and multi-scale time dependencies in traffic flow prediction, we propose a Transformer-based Hypergraph Convolutional Neural Network (TSHGCN) for traffic flow prediction. This model incorporates an efficient channel attention mechanism, a Hypergraph convolution method, and an improved Transformer time series learning module, significantly enhancing the performance of spatial feature extraction and temporal modeling.

Firstly, the efficient channel attention mechanism enhances the model’s ability to capture spatiotemporal features of traffic flow by adaptively adjusting channel weights. This mechanism significantly improves the efficiency and accuracy of feature representation without significantly increasing computational costs.

Secondly, the method based on hypergraph convolution can accurately capture the high-order dependencies between sensor nodes in the traffic network by constructing a hypergraph. This method enhances the modeling capability of complex graph structures by calculating the node degree matrix and hyperedge degree matrix, and propagating node features using the hypergraph Laplacian matrix. Compared with traditional graph convolutional networks, hypergraph convolution can represent the spatial complexity of traffic networks more comprehensively and precisely, especially showing significant advantages in handling multi-node collaborative relationships.

Thirdly, the improved Transformer module utilizes a special self-attention mechanism and distillation operation, combined with a multi-period modeling approach, to effectively capture the long-term temporal dependencies in traffic flow. This design not only overcomes the limitations of traditional methods in long-sequence modeling but also further improves prediction accuracy by integrating multi-scale temporal features.

Furthermore, the Transformer-based hypergraph convolutional traffic flow prediction model adopts a modular design concept, effectively integrating ECA, hypergraph convolution, and Transformer into spatial and temporal learning modules. This design not only maintains the high performance of the model but also significantly reduces computational complexity, making it more feasible and scalable for practical applications.

The experimental results demonstrate that the TSHGCN model outperforms existing methods across multiple traffic datasets, offering an effective and robust solution in the field of traffic flow prediction.
